# Sequence Validation of Candidates for Selectively Important Genes in Sunflower

**DOI:** 10.1371/journal.pone.0071941

**Published:** 2013-08-26

**Authors:** Mark A. Chapman, Jennifer R. Mandel, John M. Burke

**Affiliations:** Department of Plant Biology, University of Georgia, Athens, Georgia, United States of America; University of Iceland, Iceland

## Abstract

Analyses aimed at identifying genes that have been targeted by past selection provide a powerful means for investigating the molecular basis of adaptive differentiation. In the case of crop plants, such studies have the potential to not only shed light on important evolutionary processes, but also to identify genes of agronomic interest. In this study, we test for evidence of positive selection at the DNA sequence level in a set of candidate genes previously identified in a genome-wide scan for genotypic evidence of selection during the evolution of cultivated sunflower. In the majority of cases, we were able to confirm the effects of selection in shaping diversity at these loci. Notably, the genes that were found to be under selection via our sequence-based analyses were devoid of variation in the cultivated sunflower gene pool. This result confirms a possible strategy for streamlining the search for adaptively-important loci process by pre-screening the derived population to identify the strongest candidates before sequencing them in the ancestral population.

## Introduction

Identifying the molecular basis of phenotypic differentiation and understanding the role of selection in producing such differences is a major goal of evolutionary genetics [1], [2]. In the case of crop plants, strong selection is thought to have produced the remarkable phenotypic divergence that is commonly observed between wild and domesticated forms [3], [4], and identifying the causal genes has the potential to facilitate future crop improvement efforts. Numerous QTL mapping and, more recently, association studies have investigated the genetic basis of domestication-related phenotypes by testing for marker-trait associations in mapping populations [5]–[10]. While these studies have been successful in identifying numerous genomic regions, and sometimes the genes or even causal mutations influencing crop-related traits [11]–[15], such approaches have some drawbacks. For example, these methods require the development and characterization of relatively large populations and they also rely on the presence of segregating variation in order to identify genomic regions associated with a particular trait. Unfortunately, in some cases, the appropriate variation may not be available due to the occurrence of population bottlenecks and/or strong selective sweeps, and conclusions from such studies are also limited to the specific phenotypes under study.

A complementary approach to the above map-based methods is to use patterns of population genetic variation to identify putative targets of selection in the genome. Strong selection is known to influence patterns of diversity and, in the case of crop domestication, the molecular targets of selection are expected to exhibit reduced polymorphism in the crop gene pool (as compared to levels in the wild or landrace gene pools) and skewed allele frequencies relative to non-selected loci [16]–[19]. Rejection of the null hypothesis of neutrality provides evidence that the gene or region of interest has been the target of past selection. Identifying such loci through their patterns of DNA polymorphism therefore circumvents the need for creating large mapping populations and does not limit the loci detected to being involved in specific phenotypes. While this sort of approach is increasingly being applied to DNA sequence data – especially thanks to the availability of next generation sequencing technologies (e.g. [20]–[22]) – for which formal molecular evolutionary tests of selection are available, it has also been applied to large genotypic datasets [23]–[25]. In such cases, candidates for loci that have experience positive (i.e., directional) selection are often identified as those that have lost a greater than expected amount of diversity in the derived vs. ancestral populations – i.e., they fall in the extreme tail of the diversity distribution [26]–[28]. It is, however, desirable to couple such outlier-based analyses of genotypic data with sequence-based molecular evolutionary analyses as a means of validating the effects of selection and protecting against false positives (e.g. [29]).

Genotypic scans for selection have been performed in a variety of crop species [23]–[25]. In maize, for example, Vigouroux et al. [25] screened 501 gene-based simple sequence repeats (SSRs) and demonstrated strong evidence for positive selection in ten genes during domestication/improvement, making them good candidates for genes underlying agronomic traits. Similarly, Casa et al. [23] identified numerous genomic regions that may have been targeted by selection during sorghum evolution based on patterns of SSR diversity, though sequence-based analyses later failed to corroborate these findings, possibly due to the outgroup being too closely related for the ML-HKA test to be effective [30]. Because strong selective sweeps, such as those that are thought occur during domestication, are expected to cause a drastic reduction in DNA polymorphism, it is notable that two studies of maize have identified selectively important loci by first ‘pre-screening’ the derived germplasm (i.e. inbred maize cultivars) to identify loci with an absence of DNA polymorphism [22], .

In sunflower, which is a globally-important oilseed crop and also an important source of edible seeds, Chapman et al. [24] analyzed 492 gene-based SSRs in a stratified sample of wild, domesticated, and improved sunflower and identified 36 genes with evidence of selection during either domestication or improvement. Six of these genes (including three domestication-related and three improvement-related genes) were further investigated using DNA sequence-based tests for selection and the effects of selection were validated in all six cases. Here, we describe the sequencing and analysis of additional genes from this study to confirm the role of selection in shaping diversity at these loci, to better understand the timing of such selection, and to investigate, where possible, the types of variants differentiating the wild, landrace (also known as ‘primitive’ lines in previous publications), and/or improved alleles. We further argue that a pre-screening approach similar to that employed in maize (see above) would help to ‘fast-track’ the identification of loci bearing the genomic signature of selection during domestication and/or improvement.

## Methods

### Genes of interest and PCR primer design

This study focuses on 36 candidates for genes targeted by selection during sunflower domestication/improvement that were identified by Chapman et al. [24]. Six of these have previously been subjected to molecular evolutionary analyses. In the present study, we attempted to amplify portions of the 30 remaining genes from a panel of individuals (Table S1) representing eight wild, six landrace, and six improved sunflower accessions plus an outgroup (*H. petiolaris*). This was the same panel of individuals that was used to investigate patterns of DNA sequence variation in the original six genes, as well as in an analysis of selection on genes in the fatty acid biosynthetic pathway (see [24], [32]). Briefly, polymerase chain reaction (PCR) primers were designed by downloading unigene sequences from the Compositae Genome Project EST database (http://compgenomics.ucdavis.edu/), comparing them against genomic sequences from *Arabidopsis*, rice, grape, and poplar to infer the likely intron positions, and then using primer3 [33] to design primers that flanked regions spanning ca. 500–1,000 bp of coding and non-coding sequence. Due to the short length of a number of the original unigene sequences, we performed genome walking to increase the amount of sequence available for our analyses (see ref. [24]). For nine genes, we were either unable to recover sufficient sequence information via genome walking, or were unable to design primers that produced consistent amplification across both cultivated and wild sunflower. As a result, we were left with a total of 27 genes (21 sequenced herein plus the 6 from the previous study) having sufficient data for selection analyses. Based on the previously inferred timing of selection in the initial genotypic screen, these included 13 candidate domestication genes and 14 candidate improvement genes.

### Locus amplification and sequencing

Loci were amplified via PCR with each reaction containing 10 ng of template DNA, 30 mM Tricine pH 8.4-KOH, 50 mM KCl, 2 mM MgCl2, 100 µM each deoxynucleotide triphosphate, 0.1 µM each primer, and one unit of Taq DNA polymerase. PCR conditions used a touchdown protocol to minimise spurious amplification as follows: initial denaturation at 95°C for 3min; 10 cycles of 30 s at 94°C, 30 s at 65°C (annealing temperature was reduced by 1° per cycle), and 45 s at 72°C; followed by 30 cycles of 30 s at 94°C, 30 s at 55°C, and 45–90 s at 72°C; and a final extension time of 20 min at 72°C. Amplification was confirmed using agarose gel electrophoresis. Primer sequences are listed in Table S2.

PCR products were treated with 4 units Exonuclease I and 0.8 units Shrimp Alkaline Phosphatase (USB, Cleveland, OH) at 37°C for 45 min followed by enzyme denaturation at 80°C for 15 min to prepare for sequencing. BigDye v3.1 (Applied Biosystems) was used for the DNA sequencing reaction following the manufacturer's protocol, except that a reduced volume of BigDye was used in each reaction. Unincorporated dyes were removed from the sequencing reactions via Sephadex clean-up (Amersham), and the sequences were resolved on an ABI 3730xl (Applied Biosystems).

Where individuals were heterozygous for an insertion/deletion (indel), the PCR product was cloned into pGEM-T vector (Promega), transformed into competent *Escherichia coli*, and PCR-screened for the presence of an insert. Four or five positive colonies were then sequenced as above except that vector primers (T7 and SP6) were used.

### Selection analyses

Tests for evidence of positive selection were performed using the maximum-likelihood (ML) version of the Hudson-Kreitman-Aguade (HKA; [34]) test (MLHKA; [35]) as previously described [24]. Parameters required for this test were estimated for each locus using DnaSP [36]. These included the number of segregating sites (*S*), nucleotide diversity (*p*), number of haplotypes, and Watterson's [37] estimate of diversity (*θ*). In order to distinguish the loss of genetic diversity that is due to the domestication bottleneck from true events of positive selection, sequence diversity at each of the 27 genes was compared to that of the seven putatively neutral genes within the ML-HKA framework. Before doing this, however, we first tested each of the putatively neutral loci against the other six loci, as follows. First, a strictly neutral model was run, followed by a model in which each gene was compared to the other six genes. These tests were carried out separately for the wild, landrace, and improved datasets. Two times the difference in log-likelihoods of the models was then used in a Chi^­^-square (χ^2^) test with two degrees of freedom to test for statistical significance. Importantly, none of the neutral loci showed evidence of selection, establishing their validity as control loci for the investigation of selection on the candidate genes. Each of the 27 genes was then tested against the neutral loci using the approach outline above. By carrying out the tests for wild, landrace, and improved gene pools separately, we were also able to investigate the timing of selection (i.e., during domestication vs. improvement) in cases where selection was detected. The parameters employed in the ML-HKA analyses are listed in Table S3 and all previously published and newly generated sequences have been deposited in Genbank under accession numbers FJ373512 – FJ373879 and KF159030 – KF159529, respectively.

## Results and Discussion

**Figure 1:**
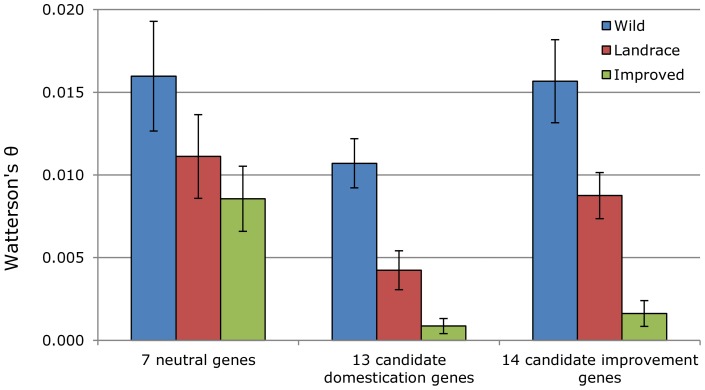
Average (± SE) genetic diversity (Watterson's θ [37]) in wild, landrace, and improved sunflower based on the sequencing of presumptively neutral genes as well as the candidates for selectively-important genes.

The process of plant domestication is predicted to result in a genome-wide reduction in genetic diversity, commonly referred to as a domestication bottleneck, in the crop gene pool as compared to that of its wild progenitor [31], [38]. A further reduction in genetic diversity can occur as a by-product of the continued narrowing of the genetic base in more highly improved varieties [3]. Superimposed on these genome-wide reductions in genetic diversity are localized losses of diversity owing to the effects of directional selection during domestication and/or improvement. As expected, both the neutral control genes and the candidates for selectively important genes exhibited the highest levels of sequence diversity (estimated here as Watterson's *θ*) in wild sunflower and the lowest levels in the improved cultivars (Table 1; Figure 1). The landraces, which represent an intermediate stage between wild sunflower and modern cultivars, exhibited intermediate levels of nucleotide diversity. Looking across classes, however, it's clear that the diversity loss in the landraces was much greater for the candidate domestication vs. improvement genes. Indeed, the domestication genes exhibited a ca. 60% loss of sequence diversity in the landraces as compared to wild sunflower vs. 45% for the improvement genes. This was, once again, expected based on how these genes were initially identified/categorized.

**Table 1:** Genetic diversity (Watterson's θ [37]) for seven neutral genes (N), 13 putative domestication genes (D), and 14 putative improvement genes (I) sampled from wild (Wild), landrace (Land) and improved (Imp) sunflower populations.

			Watterson's θ			ML-HKA *P*-values		
Type	Locus		Wild	Land	Imp	Wild	Land	Imp
N	c0025	Aleurain-like protease	0.0239	0.0150	0.0138	0.1937	0.2979	0.1760
N	c1111	Protein kinase family protein	0.0094	0.0006	0.0019	0.2125	0.6362	0.9305
N	c1351	Chlorophyll binding protein	0.0138	0.0180	0.0111	0.7964	0.7016	0.3830
N	c2016	DNAJ heat shock N-terminal domain-containing	0.0301	0.0189	0.0162	0.7361	0.8933	0.3601
N	c2307	Glyceraldehyde-3-phosphate dehydrogenase	0.0050	0.0055	0.0062	0.8146	0.7105	0.7910
N	c5369	S-adenosylmethionine synthetase	0.0111	0.0097	0.0052	0.8648	0.8339	0.7842
N	c5456	Vacuolar H+-ATPase subunit A	0.0185	0.0101	0.0054	0.2398	0.3041	0.8800
	**AVG**		**0.0160**	**0.0111**	**0.0086**			
D	c1357	Pentatricopeptide repeat-containing protein	0.0107	0.0056	0.0056	0.6688	0.3820	0.2101
D	c1533	Microtubule-associated protein	0.0216	0.0096	0.0004	0.5902	0.9072	0.0151**
D	c1649	Putative protein	0.0093	0.0087	0.0000	0.9578	0.9188	0.0029**
D	c1666¶	Putative Ser/Thr protein kinase	0.0134	0.0139	0.0000	0.1076	0.0823*	0.0452**
D	c2873	11S globulin precursor	0.0047	0.0025	0.0000	0.8921	0.5324	0.1425
D	c2963	BEL1-related homeotic protein	0.0127	0.0018	0.0000	0.8280	0.1798	0.0007**
D	c3115	Nicotinate phosphoribosyltransferase-like protein	0.0027	0.0000	0.0000	0.1256	0.9774	0.9065
D	c5898¶	Unknown protein	0.0162	0.0015	0.0008	0.7698	0.0744*	0.0269**
D	c4973¶	Chorismate synthase	0.0084	0.0000	0.0000	0.9106	0.0053**	0.0062**
D	G13K16	No significant similarity	0.0095	0.0033	0.0026	0.8837	0.2176	0.1488
D	H4B03	Kinesin-related protein (MKRP2)	0.0132	0.0030	0.0003	0.0570	0.2177	0.5660
D	M23M12	CONSTANS 3	0.0138	0.0052	0.0016	0.2596	0.2188	0.9425
D	N21O05	Thiol protease	0.0029	0.0000	0.0000	0.1685	0.0053**	0.0050**
	**AVG**		**0.0107**	**0.0042**	**0.0009**			
I	c1144	Calmodulin-binding protein	0.0024	0.0023	0.0000	0.7282	0.5471	0.0093**
I	c1236	NSL1 (NECROTIC SPOTTED LESIONS1)	0.0119	0.0068	0.0000	0.5399	0.5683	0.0051**
I	c1258	11S globulin precursor	0.0107	0.0066	0.0000	0.9099	0.4632	0.0000**
I	c1406¶	Protein kinase-like protein	0.0219	0.0194	0.0000	0.2071	0.1630	0.0050**
I	c1700	Mitochondrial dicarboxylate carrier	0.0213	0.0136	0.0084	0.8200	0.9343	0.5555
I	c1774	No significant similarity	0.0106	0.0109	0.0033	0.9305	0.8681	0.3536
I	c0019	Unknown protein	0.0250	0.0183	0.0000	0.1993	0.1024	0.0036**
I	c1921¶	Dof27	0.0154	0.0088	0.0010	0.9483	0.6381	0.0104**
I	c2150	NADP-specific glutatamate dehydrogenase	0.0321	0.0076	0.0000	0.3002	0.5340	0.0450**
I	c2588	ATIDD11 (INDETERMINATE-DOMAIN11)	0.0053	0.0087	0.0000	0.3958	0.8769	0.0017**
I	c3070	Gly-rich RNA binding protein	0.0088	0.0067	0.0077	0.9702	0.7709	0.7452
I	c5666	Peroxidase	0.0237	0.0057	0.0000	0.3859	0.4254	0.0192**
I	J22O06	Unknown protein	0.0264	0.0031	0.0000	0.3104	0.3827	0.0010**
I	L2K11	SDL-1 protein	0.0038	0.0040	0.0024	0.0879	0.0457**	0.1359
	**AVG**		**0.0157**	**0.0088**	**0.0016**			

Six previously analysed genes are indicated by ¶. *P*-values are given for the results of the ML-HKA test for each candidate gene in each of the three populations. * Significant at *P*≤0.1,**P≤0.05. Comparisons that remained significant after false discovery rate correction are indicated in bold (FDR <0.05) and underlined (0.05< FDR <0.1).

### Evidence for selection during domestication and/or improvement

**Figure 2:**
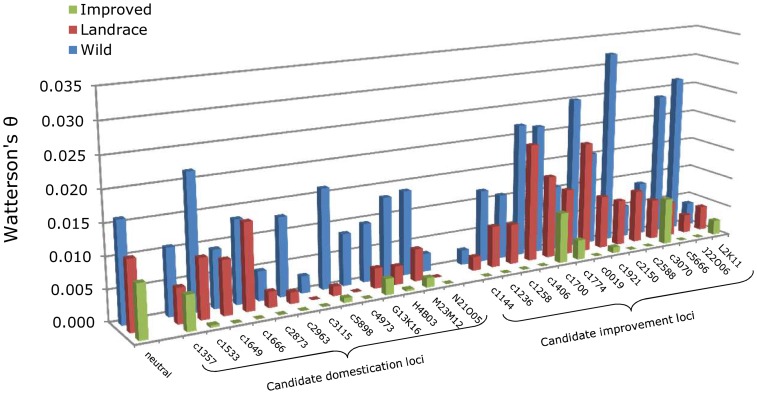
Genetic diversity (Watterson's θ [37]) in wild (blue), landrace (red), and improved (green) sunflower for 7 presumptively neutral genes (averaged), 13 putative domestication genes, and 14 putative improvement genes.

Of the 27 genes that we tested for DNA sequence-based evidence of selection during domestication and/or improvement (including 6 from our prior study; [24]), 17 (63.0%) exhibited statistically significant departures from neutrality in the ML-HKA tests (*P*<0.05) in at least one of the comparisons (Table 1; Figure 2). These 17 genes included 7 of the 13 (54%) candidate domestication genes (two with marginal [0.05< *P*<0.1] significance during that phase, but significant evidence of selection during improvement) and 10 of the 14 (71%) candidate improvement genes. Applying an FDR correction [39] using the program QVALUE (available from http://genomics.princeton.edu/storeylab/qvalue/) in the R statistics package (http://www.r-project.org/) reduced this to ten loci at FDR <0.05, including four domestication-related and six improvement-related genes, with a three additional loci exhibiting marginal significance for selection during sunflower improvement after FDR correction (0.05< *P*<0.10). In all cases, genetic diversity was severely reduced as compared to the neutral control genes in the selected population(s) – i.e., landrace and improved for the domestication-related genes or improved only for the improvement-related genes (Figure 2).

Interestingly, regardless of our initial classification of these genes, there was a tendency to detect selection more frequently during improvement vs. domestication. Thus, while our initial SSR screen suggested a roughly 50∶50 split between domestication and improvement genes, the sequence-based analyses described herein suggest a bias toward selection during improvement (Table 1). This difference may, however, be a by-product of differences in the sampling scheme between the SSR-based and sequence-based analyses. Notably, we focused our sequence-based analyses on a set of individuals from six landraces, whereas the SSR-based analyses utilized population-level sampling from a total of eight landraces. Given that the sunflower landraces are genetically quite diverse [24], [32], [40], a larger sample size in the initial analyses could have diluted the effects of more divergent landraces, resulting in significant tests in the wild-landrace comparisons in the earlier, SSR-based study but not in the present analysis of sequence diversity. In this context, it is worth noting that for three of the genes the showed evidence of selection during improvement in the current study (c1258, c1533 and c2963), the Maiz Negro landrace harbors an allele that was divergent from all other landrace and improved lines. Re-analysis without this line resulted in significant tests for selection during domestication for c1258 and c2963 (*P*≤0.001). For c1533, the outgroup allele only exhibited one SNP relative to the most common allele in cultivated sunflower, potentially impacting our ability to detect selection. Similarly, in the study of sorghum domestication referenced above, low divergence of the out group from sorghum was one of the reasons given for the small number of loci that showed departure from neutrality [30].

While our analyses provide clear statistical evidence of the role of selection in shaping sequence diversity in a number of genes, it must be kept in mind that the effects of selective sweeps can extend into linked, neighbouring regions. It thus remains possible that the genes showing evidence of selection are linked to the actual targets of selection as opposed to having been targeted by selection themselves. In this light, it is worth noting that the initial studies of linkage disequilibrium (LD) in sunflower found evidence for relatively rapid decay [41], [42], suggesting that positive signatures of selection should be very tightly linked to the targeted variants. More recently, however, evidence of localized islands of extended LD has emerged [9] and selection targeting a fatty acid desaturase gene has been shown to have resulted in a sweep spanning ≥100 kb [32]. As such, the genes identified herein as showing evidence of positive selection during the evolution of cultivated sunflower may simply be demarcating selectively important genomic regions. A better understanding of the functional significance of these genes awaits further investigation and/or experimentation.

For the loci with significant evidence of selection after applying the FDR correction, we identified SNPs that differentiated the alleles in different gene pools, specifically looking for what appeared to be novel variants or fixed, non-synonymous differences. Two loci (c0019, and c5666) exhibited at least one fixed non-synonymous mutation in the improved gene pool that was found to be at low frequency (<20%) in the wild. Two additional loci (c1649 and c2963) had at least one non-synonymous polymorphism (and several non-coding variants) that showed fixed differences between the wild and improved gene pools. Finally, for one locus (c5898), a single cultivated line (RHA801) contained an amino acid insertion that was not present in the sampled landrace lines, but was present at low frequency in the wild, possibly suggesting introgression from the wild into this line. While it is possible that some of these non-synonymous differences could be adaptive, it must be kept in mind that these findings are based on relatively limited sampling and that we also lack data from the full lengths of these genes. As such, care should be taken to avoid reading too much into these results.

As for why a subset of the loci identified as being under selection in the original SSR screen did not show evidence of selection at the sequence level, it should be kept in mind that the tests employed in that study were not, for the most part, formal molecular evolutionary analyses. Rather, they were largely based on the identification of extreme outliers, an approach that may have been more prone to false positives. Also, as noted above, the sequence-based tests for selection employed smaller sample sizes. As such, one or two highly divergent alleles could produce a non-significant ML-HKA test result, whereas this effect could have been diluted in the larger screen of SSR polymorphism.

### Increasing the efficiency of screens for selection

In addition to confirming the effects of selection on population genetic diversity at the majority of loci that we had previously identified as bearing the signature of selection in sunflower, our results also provide methodological insights. Our results highlight a potential means for increasing the efficiency of sequence-based screens for selection in a pool of candidate genes. Because all 10 genes that showed sequence-based evidence of positive selection were devoid of sequence variation in the selected population(s), it should be possible to enrich for selectively important loci by performing a pre-screen of the derived population to identify loci with exceptionally low levels of diversity. This subset of loci can then be assayed in the ancestral population to produce the data necessary for formal tests of selection. In fact, this general approach has been successfully applied in two studies of maize [22], [31]. Our results in sunflower suggest that it may be generally applicable to studies of crop domestication.

## Supporting Information

Table S1 **Accessions from which the individuals employed in the DNA sequence analyses were sampled.**
(DOCX)

Table S2 **Polymerase chain reaction (PCR) primer sequences.**
(DOCX)

Table S3 **Extension of** **Table 1** **to contain the parameter estimates used in the ML-HKA test.**
(XLSX)
