# Supplementary material for: Sequence Validation of Candidates for Selectively Important Genes in Sunflower
Source: PLoS One. 2013 Aug 26;8(8):e71941. doi: 10.1371/journal.pone.0071941 (PMC3753318; doi:10.1371/journal.pone.0071941)
Supplement: Table S1 — Accessions from which the individuals employed in the DNA sequence analyses were sampled. (DOCX) [file pone.0071941.s001.docx]

**Table S1 –** Accessions from which the individuals employed in the DNA sequence analyses were sampled.

| **Name** | **Status** | **Collection Locale** | **PI Number^a^** |
| --- | --- | --- | --- |
| Ames14400 | Wild | Arizona | 649851 |
| Ann-1114 | Wild | Arkansas | 613727 |
| A-1473 | Wild | Kansas | 413027 |
| A-1572 | Wild | Mexico-Mayo | 413123 |
| A-1455 | Wild | Missouri | 413011 |
| Ames23238 | Wild | Ohio | 649853 |
| Ames23940 | Wild | South Dakota | 649854 |
| Ann-646 | Wild | Tennessee | 435552 |
| Havasupai | Landrace | n/a | 369358 |
| Hidatsa | Landrace | n/a | 600721 |
| Hopi | Landrace | n/a | 432504 |
| Maiz Negro | Landrace | n/a | 650761 |
| Mandan | Landrace | n/a | 600717 |
| Seneca | Landrace | n/a | 369360 |
| RHA280 | Improved | n/a | 552943 |
| RHA801 | Improved | n/a | 599768 |
| cmsHA89 | Improved | n/a | 650572 |
| Mennonite | Improved | n/a | 650650 |
| Pervenets | Improved | n/a | 483077 |
| VNIIMK1646 | Improved | n/a | 650385 |
| *H.petiolaris* | Outgroup | Texas | 435829 |

^a^See http://www.ars-grin.gov/npgs/index.html
