# Supplementary material for: Sequence Validation of Candidates for Selectively Important Genes in Sunflower
Source: PLoS One. 2013 Aug 26;8(8):e71941. doi: 10.1371/journal.pone.0071941 (PMC3753318; doi:10.1371/journal.pone.0071941)
Supplement: Table S2 — Polymerase chain reaction (PCR) primer sequences. (DOCX) [file pone.0071941.s002.docx]

**Table S2 –** Polymerase chain reaction (PCR) primer sequences.

| **Locus** | **Forward (5’ – 3’)** | **Reverse (5’ – 3’)** |
| --- | --- | --- |
| N21O05 | GTTTTTACAAATGGATTAGGGT | CCGCAACAACCATACATGAAGCAA |
| c3115 | TAAGGGGACGGAGATTACAAA | GATGAACCTTATGCACTCTTCAA |
| H4B03 | GATGAGCTGGTATGGGACAATGG | TGCACCCAAAAGTAAAGCAGGGC |
| c1533 | AGTCATGGAWAGCCCAAGATTAA | CAAATAATGGCATCAGCTGAGCT |
| c1357 | GAAGGCTGGAAATTCTACAACA | TTCACTAATAACCTCTGCAAGAACA |
| c2873 | TTGTTCATCACCATCCCATTTA | GGAATTTGAAGCTGTTGAGGTT |
| c2963 | GGGCTTAGGAGAGGAAGACAAT | TGTTGCTAGAATACGGTGGTTG |
| M23M12 | TTCATGTACAATTTCACGTCTCC | CACAACATACATGATGAGATACAACA |
| G13K16 | GATGCACTGCAAGAAATGAGTC | ATGCACATCTTGTTCACCATCA |
| c1649 | TTCAATGGACATCGAAAAGAAA | TTCAATAGTCCAACGTATAACATAACA |
| c1700 | GCCAGCCCAAATTACTGAATTAG | CCCCAAAAACACAATCAAAAATCC |
| c2150 | GTTACCCAACCTATCCGATCCGC | CCTTGTTCTGACATACTCGTAG |
| c1774 | GTAAAGGTGGACCGGGTGGTCAA | CCGATTAGTACGGAATAACATA |
| c0019 | GGATGTTGATGTATAAGCAGAA | ATTGCATCCAATCAAGCATAGC |
| c2588 | TAAATCTTTGAATAAATAGGATA | TGATGAYGGTGATGAACAAACCA |
| J22O06 | CGATGGTTTTCATATGGGTTTTAT | AAAGAATTCTCCTGCAAATGGACC |
| c1144 | TCAAGAAGGATACGTGTACTGG | CATGTCATGCATTCCAACTTCAC |
| c5666 | GTATGCGGTGGAGCTAGGAAGGTT | CCAACWCGRCCTAGTTTAGTCATA |
| c3070 | AGCATCCTCTCAATCCACAAAT | GGAGTAACGAGACCCACCATC |
| L2K11 | TCAAGAAGAAYTATGACCATCTTCC | ATTCCCTTCCTCATGAGTTTCA |
| c1258 | CAGCAACAACATCAGCAGCAACAG | GATTCCTCATCGTACTCGGACATT |
